# Prediction of Center-of-Mass Kinematics of Sensopro Exercises with Neural Network Models

**DOI:** 10.3390/s26103051

**Published:** 2026-05-12

**Authors:** Heinz Hegi, Michael Single, Tobias Nef, Ralf Kredel

**Affiliations:** 1 Institute of Sport Science, University of Bern, 3012 Bern, Switzerland; 2 Gerontechnology and Rehabilitation Group, ARTORG Center for Biomedical Engineering Research, University of Bern, 3010 Bern, Switzerland; michael.single@unibe.ch (M.S.); tobias.nef@unibe.ch (T.N.)

**Keywords:** coordination training, center of mass, deep learning, time-series analysis

## Abstract

Monitoring center-of-mass is crucial for assessing postural control, but field measurements are often impractical or cost-prohibitive. This study investigates the feasibility of predicting center-of-mass kinematics from the motion of an unstable base—the Sensopro Luna—using deep learning, eliminating the need for wearable sensors. We conducted a cross-sectional study in which 64 participants were recorded performing three coordination exercises (Single-Leg Stance, Stepping, and Waves). Marker-based motion capture and auxiliary inertial sensors were used to record reference and tape kinematics. The model inputs consisted of IMU- and motion-capture-derived tape segment orientations, IMU accelerations and angular velocities, and algorithmic estimates of the lowest tape positions. Nine axis-specific exercise models were developed using a hybrid Encoder–LSTM–Decoder architecture and compared against linear regression baselines. Our results indicate that the deep learning models successfully predicted horizontal center-of-mass displacements (DNN Mean Absolute Errors of 16.1–23.7 mm for X-axis and 4.4–31.3 mm for Y-axis) and exhibited descriptively lower errors than linear models in mean absolute error and signal morphology. However, vertical predictions were less reliable, likely due to the physical constraints inherent to the kinematics of the unstable base. Error analysis revealed that prediction accuracy was highest within common postural ranges, but decreased for extreme displacements. These findings provide a proof-of-concept for wearable-free postural monitoring, particularly for movement along the mediolateral and sagittal axes. Such a system could facilitate automated, cost-effective postural feedback and performance tracking in rehabilitation and fitness environments, supporting autonomous coordination training without the practical constraints of traditional measurement systems.

## 1. Introduction

Postural control is essential for maintaining the postural stability required for the execution of accurate, functional movements during sporting activities and activities of daily living [1]. Without proper stimuli, prolonged inactivity and age can lead to a decline in postural control, increasing the risk of falls and potentially creating a negative feedback loop of increasingly sedentary behavior [2,3]. Balance and coordination training—such as exercising on unstable support surfaces—is one of several strategies to combat these issues [4,5]. Demanding and dynamic training conditions that require continual postural adjustments provide a strong stimulus to preserve or improve postural control over time [6]. Importantly, unstable bases provide a defined training area well-suited to stationary sensor systems, which facilitate automated performance assessments and feedback, such as center-of-mass (CoM) kinematics [7,8]. CoM is a fundamental biomechanical parameter in postural control assessment. It provides critical insights into stability during both static and dynamic activities across diverse applications, including clinical diagnostics and rehabilitation, sports performance evaluation, and fall risk assessment in older populations [9,10,11]. Despite its wide use, practical CoM monitoring outside laboratory settings remains challenging.

Most prior work has examined movement tasks on stable bases and derived postural control variables from wearables or rigid force plates [12,13,14,15,16]. For quantifying movements on unstable bases of support, utilized measurement systems included wearable IMUs [17,18,19], depth cameras for pose estimation [20,21], marker-based motion capture systems [7,8,22], and force plates or cameras below the support surface [23,24,25]. While IMUs directly attached to an unstable base of support have also been investigated before, these aimed for markedly different feedback variables: Singh et al. used an accelerometer attached to a slackline to construct and evaluate gait phase classifiers [26], and Zadrapova et al. instrumented a wobble board with a single IMU to provide tilt feedback during training [27]. To our knowledge, this is the first work that explores whether the motion of an unstable base itself can serve as a proxy for CoM kinematics without the use of body-worn sensors. Although existing optical motion capture systems for estimating CoM kinematics have high accuracy [28], they typically require expert supervision and expensive camera systems unsuitable for field conditions. Alternative systems based on wearable Inertial Measurement Units (IMUs) have also been proposed [11,29]. However, wearables impose user setup, calibration, and compliance burdens that limit scalability in gyms and clinics.

Here, we avoid these limitations by leveraging kinematic measurements of the unstable base to predict CoM displacement without wearables. The reaction forces on the unstable base are intrinsically linked to the forces applied to the body CoM and therefore to CoM kinematics [8], provided that the base is the sole contact point with the ground and external handholds are not used. We investigate the potential of CoM estimation by using IMUs attached to the Sensopro Luna, a commercially available device comprising two elastically suspended tapes used for balance and coordination exercises. Because the mapping from the kinematics of an elastically suspended tape to the human CoM is highly nonlinear and time-dependent (owing to the complex interplay between the involved tape kinematics, tape kinetics, dampening effects, and human joint articulations), we hypothesized that nonlinear deep temporal modeling would be required to capture these dynamics accurately, prompting a comparison between linear regression and DNN approaches. If validated, such a system would enable cost-effective tracking of features related to postural control under field conditions; this could lay the foundational groundwork for future automated postural feedback and performance assessment for semi-automated balance and coordination training in therapy and fitness centers. This could help users increase self-efficacy, improve exercise execution, and undertake autonomous coordination training [30,31,32,33].

The objectives of this article are therefore (1) to implement a proof-of-concept to demonstrate that CoM position can be predicted from tape kinematics with deep neural network models; (2) to compare the relative accuracy of these models for different Sensopro exercises in all three principal spatial axes; and (3) to compare the prediction accuracy of our deep learning models with baseline linear regression models across multiple complementary performance metrics. By doing so, we aim to establish the feasibility of wearable-free CoM monitoring as a foundation for automated balance assessment and training feedback.

The remainder of this paper is organized as follows: Section 2 describes the experimental setup, data processing, model development, and statistical evaluation; Section 3 presents the prediction results for all exercise-axis combinations, including qualitative trajectory comparisons, MAE distributions, and position-dependent error analyses; Section 4 covers the main findings, limitations, and future directions; and Section 5 summarizes the feasibility of using Sensopro tape kinematics for wearable-free CoM feedback.

## 2. Materials and Methods

### 2.1. Participants

Participants for this cross-sectional study were sport science students aged 18–24 years. To control for fatigue effects and asymmetrical exercise bias, participants were allocated to eight groups of 7–9 participants that differed in exercise order and leg placement for asymmetric movements. To achieve a uniform target sample of 64 participants, 77 individuals were recruited and tested over a six-month period. Thirteen recordings were subsequently excluded due to technical issues (missing markers or system failures), resulting in a final dataset of 64 participants (33 male, 31 female). This study was conducted in accordance with the Declaration of Helsinki and was approved by the Institutional Ethics Committee of the Faculty of Human Sciences at the University of Bern (approval number: 2019-08-00004). Written informed consent was obtained from all individual participants included in the study.

### 2.2. Devices and Setup

**Figure 1:**
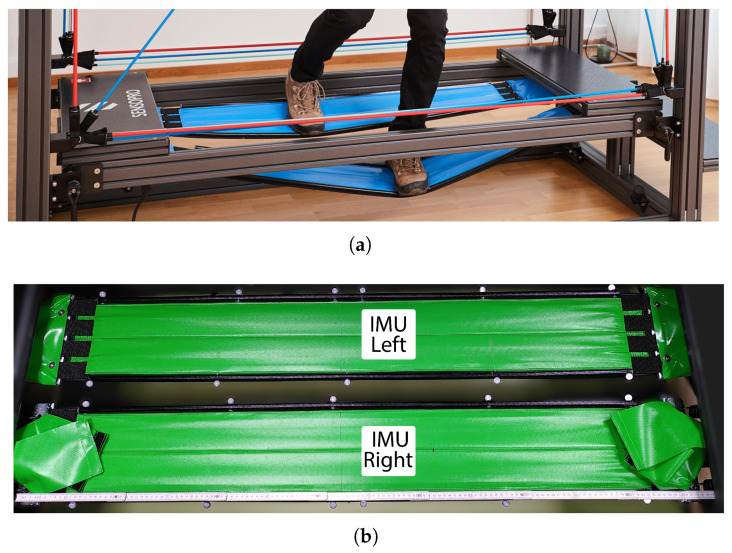
The Sensopro Luna during a sideways exercise (**a**), and tapes (**b**) showing marker and IMU placement. Copyright 2025 by Sensopro AG.

Exercises were performed on a Sensopro model Luna Fitness (Sensopro AG, Münsingen, Switzerland), as shown in Figure 1. Tape motion and body kinematics were recorded with a marker-based motion capture system (10 Vicon T20s cameras, 2 MP, 500 Hz, Vicon Nexus 2.12, Vicon Motion Systems Ltd., Oxford, UK). Tape kinematics were additionally recorded using two IMUs (Blue Trident IMUs, 500 Hz, Vicon Motion Systems Ltd., Oxford, UK) attached to the center of each tape. Anthropometric data were gathered for each participant before performing static and dynamic calibrations of the Vicon Plug-in Gait full-body model.

The inputs to our models were constructed using a combination of inertial and optical data. The central segment’s acceleration, angular velocity, and spatial orientation were captured by the IMUs (thereby avoiding signal noise from numerical differentiation [34]), yielding nine features per tape. Concurrently, the three-dimensional orientations of the anterior and posterior tape segments were extracted via the motion capture system. We then calculated the estimated coordinates of the lowest point on each tape, along with their relative vertical offset, using the geometric principles established by Hegi et al. [35]. Consequently, the final feature vector comprised 37 distinct inputs used to map to the three-dimensional CoM target.

### 2.3. Experimental Protocol

Participants first performed each of the eight selected exercises (see Appendix A) for 30 s as a warm-up. After the warm-up phase, each exercise was repeated four times in consecutive 45-s trials, with a break of at least 20 s between trials. Participants could request longer breaks if necessary. Representatives from Sensopro AG advised on the choice of exercises to ensure that they were representative of general use of the Sensopro Luna; exercises involving complex hand movements or other elements of the Sensopro (e.g., requiring holding onto elastic tubes or metal rails, other than for safety) were excluded. To maintain experimental tractability while covering a representative range of distinctive movement characteristics, we selected a subset of three exercise types for training and testing of our neural network models: (1) Single-Leg Stance, a challenging balance task, during which participants frequently needed to grab the metal rails for support; (2) Stepping in place, an exercise consisting of slow, asymmetrical movements; and (3) Waves, an exercise consisting of fast, symmetrical movements. The complete set of exercises is provided in Appendix A (Table A1).

### 2.4. Data Processing

The built-in functions of the Vicon Nexus software 2.12 were used to first fill gaps in the marker trajectories and then generate reference CoM trajectories. Trials containing less than 20 s of valid CoM data were excluded from training and analysis; this was only necessary when a participant’s posture during exercise resulted in persistent marker occlusions. To reduce potential distortions in the input orientation data due to small shifts in marker positions on the tapes or the exact Sensopro frame positioning, orientation data from the front and rear tape segments and IMUs were normalized based on static recordings taken immediately prior to the warm-up of each participant (i.e., data processing relied on brief recordings of the unloaded tapes to derive absolute orientation data from relative rotations). The orientation was converted to roll, pitch, and yaw following the intrinsic XYZ convention for Tait-Bryan angles [36]. Then, the position estimates for the lowest point on both tapes and the height difference between tapes were derived from the respective front and rear segment data using a previously validated tape model [35]. A 25 Hz low-pass Butterworth filter (fourth-order, zero-lag) was applied to all recordings. The cutoff frequency was selected so that 95–99% of the signal power was maintained [37].

### 2.5. Model-Based Prediction of CoM

To predict three-dimensional displacement (X, Y, Z) of the body’s CoM from fixed-length sequences of multivariate sensor data, we developed a deep neural network (DNN) architecture and compared its performance against a linear regression baseline. Both models processed normalized time-series signals structured as tensors.

Nine models were trained for each approach (DNN and linear regression), each specialized for one combination of exercise type (Single-Leg Stance, Stepping, Waves) and spatial axis (X, Y, Z), resulting in 18 models total. Rather than predicting absolute CoM position, the models estimated relative displacement; this accounts for the fact that absolute CoM is constrained by person-specific anthropometric features that cannot be inferred from tape kinematics alone.

All models were implemented in Python 3.11 using PyTorch (v2.5.1) and scikit-learn (v1.6.1). A complete list of dependencies and version specifications is available in the project repository [38].

#### 2.5.1. Data Partitioning

Each participant performed four trials per exercise. For each participant, one trial was randomly selected for testing, while the remaining three were assigned to the training and validation dataset. This random allocation ensured that the test set was not systematically biased by trial-order effects, such as fatigue or learning adaptations.

One trial per participant was reserved for testing (25% of the data). The remaining 75% of trials were split into training and validation subsets using an 80/20 ratio, resulting in an overall partition of 60% training, 15% validation, and 25% testing. Because this evaluation strategy tests on unseen trials from participants already represented in the training set, it assesses intra-subject prediction rather than generalization to unseen users. This approach was chosen to establish baseline algorithmic feasibility, though it remains a primary limitation regarding immediate real-world deployment.

#### 2.5.2. Neural Network Architecture

**Figure 2:**
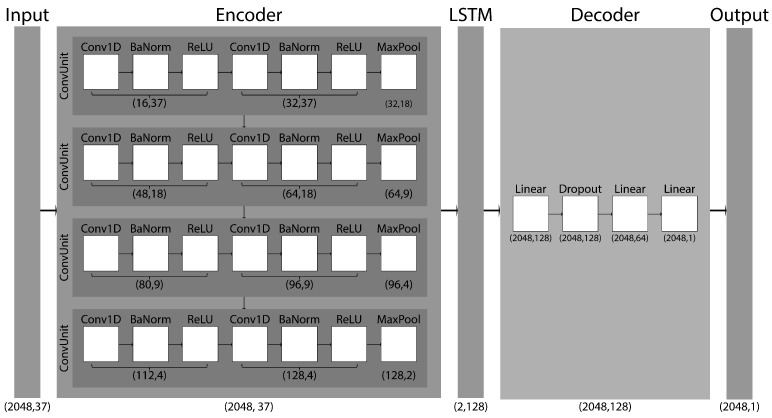
Architecture of our deep neural network for predicting three-dimensional center-of-mass (CoM) displacements: The model processes multivariate sensor input (37 input channels with a sequence length of 2048 samples) through an encoder–decoder architecture: (1) a four-block convolutional neural network (CNN) encoder extracts spatial-temporal features with progressive channel expansion and convolving features; (2) a two-layer long short-term memory (LSTM) captures temporal dependencies from the CNN features; and (3) a three-layer multilayer perceptron decoder transforms the final LSTM hidden state into 2048 displacement values representing the corresponding to the target CoM component.

We implemented a hybrid one-dimensional Encoder–LSTM–Decoder architecture to model temporal sequences and perform 1D sequence-level regression (Figure 2).

The encoder comprised a deep convolutional neural network (CNN) for feature extraction, followed by a long short-term memory (LSTM) module to capture temporal dependencies. The decoder consisted of a three-layer multilayer perceptron component that mapped the LSTM output to the final regression output. The network takes an input tensor of shape (T, $C_{in}$), where $C_{in}$ is the number of input channels (here 37) and T is the sequence length. We chose a heuristic of T = 2048, corresponding to 4 s of measurements, which captured roughly two cycles of the dynamic exercises and characteristics of postural sway during the Single-Leg Stance. Feature extraction was performed by four convolutional blocks (i.e., ConvUnit), each consisting of two convolutional (i.e., Conv1D) layers (kernel size 3, stride 1, padding 1). Each was followed by batch normalization (i.e., BaNorm), rectified linear unit (i.e., ReLU) activation, and a subsequent max-pooling (i.e., MaxPool) layer (kernel size 2, stride 2) that halved the feature resolution. The convolutional channel widths increased across blocks from 1→32→64→96→128, resulting in a downsampling factor of 16. The CNN output was then transposed to match the LSTM input format and passed to a unidirectional, two-layer LSTM with a hidden size of 128 and inter-layer dropout of 0.3, from which the final hidden state was selected as the sequence representation. This representation was passed through a three-layer fully connected regression head consisting of a 128-unit layer with ReLU activation (i.e., Linear) and dropout of 0.4 (i.e., Dropout), followed by a 64-unit layer with ReLU activation, and a final linear layer outputting a single scalar value. Batch normalization was applied after each convolutional layer, and ReLU activations were used after each convolutional and intermediate fully connected layer.

#### 2.5.3. Model Training

Models were trained using the Adam optimizer with a learning rate of 0.001 and mean squared error (MSE) as the loss function. Training was conducted for 20 epochs. Hyperparameter configurations, including sequence length, hidden sizes, and dropout rates, were selected based on heuristic pilot testing rather than exhaustive grid search optimization, serving as a baseline proof-of-concept configuration. The validation set was used to monitor training progress and prevent overfitting.

### 2.6. Statistical Analysis

Model performance was evaluated on the test set by comparing predicted CoM displacements with reference values obtained from the Vicon motion capture system.

First, we plotted model predictions with corresponding reference measurements for qualitative comparison. For visualization of the nine deep learning models, we used data from a representative participant whose prediction error was closest to the group mean.

Next, for each of the 18 trained models, we calculated mean absolute error (MAE) between predicted and reference displacement values across all time points within the corresponding trials. Results were visualized using raincloud plots combining distribution density, boxplot statistics, and individual data points. For each exercise-axis combination, linear regression and DNN models were plotted side-by-side to facilitate direct performance comparisons.

To characterize systematic, position-dependent biases in CoM prediction, we computed the prediction error as the difference between model-predicted CoM positions and the reference measurements. This analysis was performed for all nine exercise-axis combinations. All trials within each exercise-axis combination were concatenated to form error vectors. Prediction errors were then grouped according to their corresponding reference CoM positions using 80 histogram bins uniformly distributed across the range [−200,200] mm (bin width: 5 mm). Within each bin, we computed five percentile-based statistics of the error distribution: the 2nd and 98th percentiles (defining a 96% confidence interval), the 25th and 75th percentiles (interquartile range), and the 50th percentile (the median). We visualized these position-dependent error distributions and the normalized sample distributions to examine systematic deviations across spatial positions.

Finally, we computed the mean (M), the coefficient of determination ($R^{2}$), and the root mean squared error (RMSE) to quantify overall prediction accuracy and characterize model performance. The resulting RMSE values were subsequently compared with the standard deviation of their corresponding reference measurements. We used the following definition to calculate the coefficient of determination:

(1) $$R^{2}=1-\left(\frac{{\mathrm{RMSE}}^{2}}{\mathrm{Var}(y)}\times \frac{N}{N-1}\right)$$

Here, *N* denotes the total number of observations in the dataset, and Var(y) represents the sample variance, which measures the dispersion of the true target values (i.e., the reference system measurements) around their mean.

## 3. Results

In total, we recorded 256 Step, 256 Wave, and 256 Single-Leg Stance trials. Of these, three Wave trials were excluded because of missing CoM data.

### 3.1. Qualitative Plots

**Figure 3:**
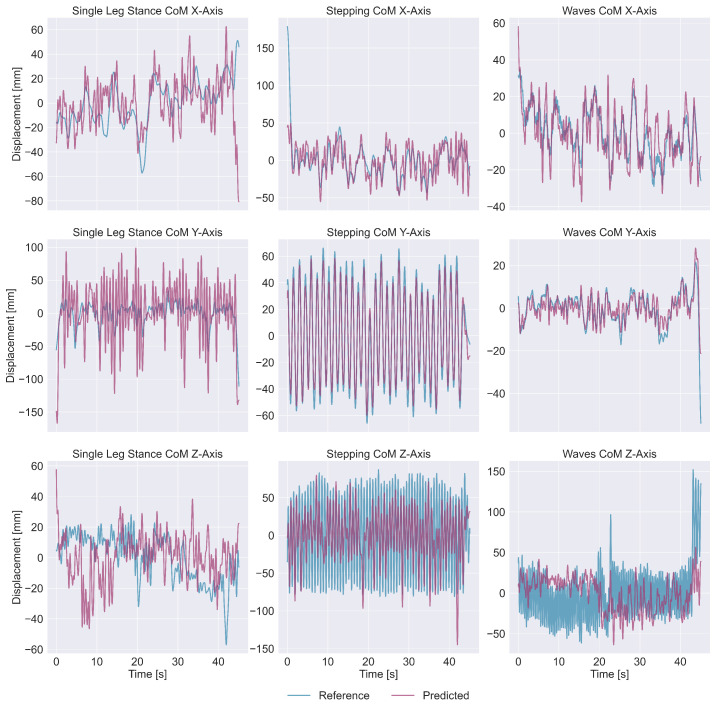
Visualization of one predicted trajectory (purple) and the corresponding reference measurement from the Vicon system (blue) along the X, Y, and Z axes for all three exercise types. The three examples shown are from representative participants whose performance was close to the average prediction error.

Qualitative comparisons between model predictions and reference measurements are illustrated for representative participants whose performance was close to the average prediction error (Figure 3). Predicted signal morphologies closely matched the reference measurements for the X- and Y-axes for all exercise types, except for the Y-axis in the Single-Leg Stance exercise. For the Z-axis, the predicted signal morphologies deviated from reference values across all exercise types.

As shown in Figure 3, the predicted signal characteristics were generally well-represented for the longitudinal (X) and lateral (Y) axes. However, prolonged deviations between the signal and prediction occurred during the Single-Leg Stance, and predictions for the vertical (Z) axis generally failed to detect sustained vertical CoM changes, instead returning to baseline equilibrium.

### 3.2. Mean Absolute Errors

**Figure 4:**
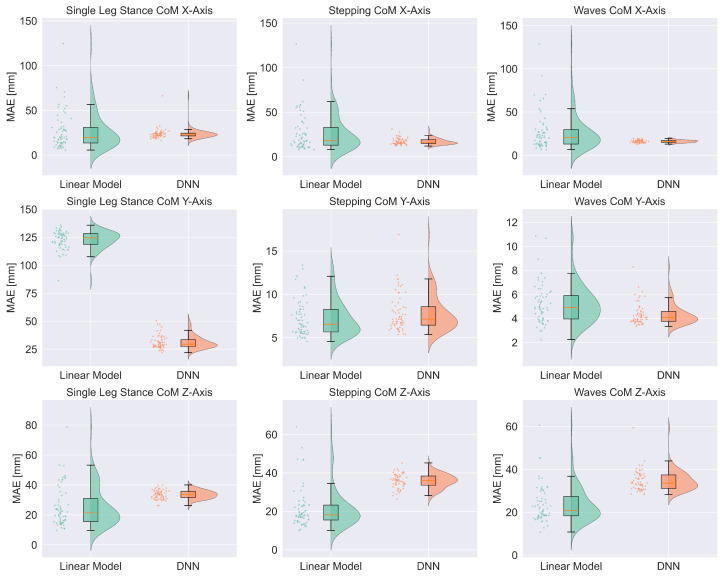
Raincloud plots illustrating the distribution of mean absolute error (MAE) values comparing the performance of the linear and deep neural network (DNN) models. Each dot corresponds to the MAE of a single participant across the 18 evaluated models.

Figure 4 shows the mean absolute errors for all trained models in the form of raincloud plots. MAE values were computed individually for each participant and for every exercise-axis combination. Furthermore, Table 1 serves as a comprehensive summary comparison, directly contrasting the performance metrics (Mean, SD, Min, Max, Median) of the linear baseline models against the DNN models across all nine exercise-axis combinations.

Figure 4 and Table 1 demonstrate that the DNN models yielded descriptively lower or similar mean absolute errors compared to the linear models for the horizontal axes (X and Y), indicating that the non-linear network better captured the complex base-to-CoM mapping. The only exception was the Stepping CoM Y-axis model, which was marginally less accurate than the linear model. Conversely, the linear models appeared to yield more consistent results than DNN models for vertical CoM position, supporting the conclusion that the system’s physical characteristics are insufficient for reliable vertical CoM position estimation.

### 3.3. Prediction Errors

**Figure 5:**
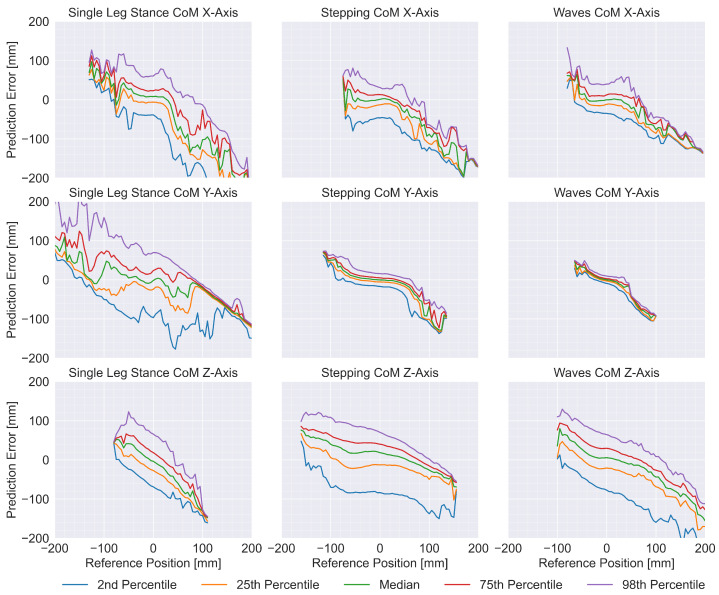
Position-dependent error distributions for each exercise and each axis. Each plot consists of five percentile curves (2nd, 25th, 50th, 75th, 98th) showing how the prediction error distribution (Y-axis) is affected by the ground truth center-of-mass (CoM) position (X-Axis).

Figure 5 shows the position-dependent error distributions, revealing systematic biases—indicated by non-horizontal median lines—and heteroscedastic error patterns presenting as varying percentile band widths across the measurement ranges. Notably, the relatively flat regions towards the center of the plots indicate that the model predictions were most reliable for smaller, more central CoM displacements.

**Figure 6:**
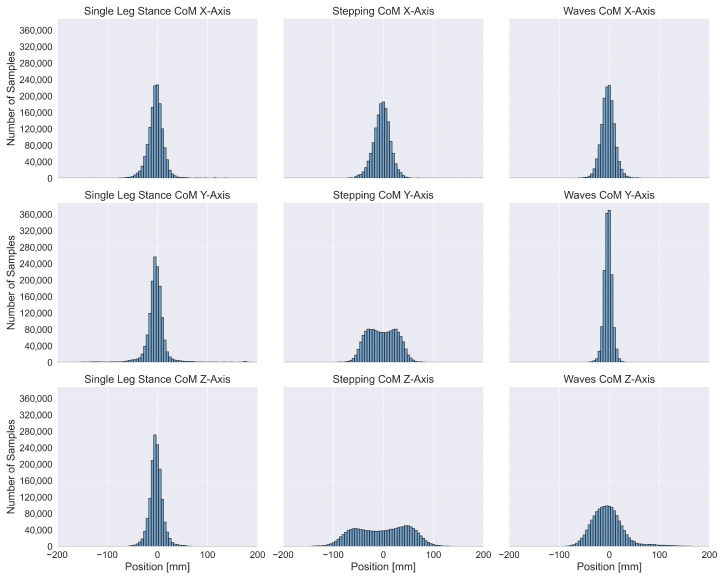
Normalized sample distributions split into different bins determined by ground truth positions. These bins form the basis for the visualization of position-dependent errors in Figure 5. All distributions resembled a normal distribution, except for the stepping CoM Y-Axis and Z-Axis.

Normalized sample distributions of the position-dependent errors are shown in Figure 6. Most sample distributions appear approximately normal across bins, but the available sample density sharply declines for larger CoM displacements. This underlying distribution largely explains the error patterns observed in Figure 5, suggesting a connection between decreased prediction robustness at the spatial extremes and insufficient training data for uncommon, extreme postural configurations.

Table 2 summarizes performance metrics of the DNN model. The RMSE of the prediction was smaller than the SD of the corresponding reference measurements for most exercise-axis combinations, indicating that the prediction error was smaller than the variability observed in the reference data. The mean value provides insights into the systematic bias in our models. The X- and Y-axis models showed moderate performance ($R^{2}=0.30$–0.78), with RMSE values below the corresponding reference SD. Z-axis performance was weakest, particularly for Single-Leg Stance ($R^{2}=0.11$) and Stepping, which showed the largest RMSE (42.4 mm). Mean errors were generally small for X/Y, while the largest bias occurred for Stepping Z-axis (17.6 mm).

## 4. Discussion

In this proof-of-concept study, we provide initial evidence for the feasibility of predicting CoM trajectories from tape kinematics during coordination exercises using DNNs.

We collected a large dataset of motion capture and IMU data from 64 participants who performed exercises on the Sensopro Luna. Using this dataset, we trained and tested one DNN and one linear regression model for each of nine different axis-exercise combinations. The DNN architecture was designed to learn relevant connections between different inputs and incorporate physical characteristics of the system, rather than just capture movement harmonics and correlations. Overall, model performance varied widely across axes, with the DNN yielding descriptively lower errors than the linear regression model (Figure 4 and Table 1). This finding serves as a proof of concept, suggesting that a deeper investigation of different DNN architectures and input combinations is worthwhile to improve CoM prediction. By employing a DNN, we bypass the need for traditional biomechanical modeling, which would be prohibitively complex in this specific application. In contrast to previous research on measurement systems for training on unstable bases of support, this work utilized kinematic measurements of the unstable base of support to predict CoM positions, which constitutes a relevant postural feedback parameter that cannot be measured by a simple sensor system directly.

While other architectures tend to use encoders to downsample and filter high-frequency signals, ours instead convolved the different inputs together. One potential disadvantage of this choice is that noisy input signals could lead to inaccurate or noisy predictions, such as the deviations observed in Figure 3. However, since the marker data had already been processed by the motion capture software, noise sensitivity should only affect the IMU data, reducing its impact on our results. Conversely, while applying a low-pass filter and downsampling the input signals would likely make sense in practical applications, we decided to omit this step and include the added data points for training data augmentation. Moreover, downsampling with a CNN would lead to an unacceptably low temporal resolution within just five layers, so adequate tracking of quick CoM movements would require an additional interpolation step. Instead, an LSTM module was used for optimal signal filtering and temporal analysis. By training separate models for each axis, we reduced task complexity and prevented high-error vertical (Z) predictions from adversely affecting the optimization of the more predictable horizontal (X/Y) trajectories.

By convolving across distinct input signals rather than temporal windows, the encoder prioritizes identifying cross-feature correlations before consolidating information over time. While we did not verify this with formal ablation studies, we hypothesize that—by convolving across distinct input signals—this functionally emulates explicit forward modeling by first deriving instant reactive forces from the unstable base, then integrating position and velocity data to estimate the current state. Overall, we expect the advantages of this architecture to outweigh the disadvantages in this context. Even so, if less reliable sensors are used in practice, a deeper investigation of alternative architectures and sensor-specific filtering strategies would likely yield better accuracy.

The qualitative plots in Figure 3 show varying prediction robustness in different cases. The prolonged deviations from the reference signal in Single Leg Stance exercises are likely due to only one tape being used in this exercise, effectively halving the relevant input. Large errors at the start and end of exercises may stem from reduced temporal context or rare transition configurations in the training data. Notably, the Waves CoM Z-axis subplot illustrates a general failure to detect sustained vertical CoM changes: standing up shifts the reference signal to a new equilibrium, but the prediction does not follow. Physically, sustained vertical CoM shifts can be produced by temporary changes in force, after which the tape constellation may revert to its original configuration; meanwhile, sustained weight shifts in the X/Y-axes induce stable changes in the tapes due to the interplay between CoM and center of pressure displacements [13,15]. Therefore, estimating absolute vertical CoM would plausibly require double integration of these transient forces over time, a task inherently more complex than the direct position-to-constellation mapping possible for X/Y. As such, the problem of vertical CoM prediction is highly ill-posed and practically infeasible using only the current steady-state features. Ultimately, this constitutes a theoretical observability constraint, rather than mere model deficiencies, which fundamentally limits the achievable Z-axis accuracy. This aligns with the stronger performance observed for X/Y.

Consistent with the qualitative plots, Figure 4 and the descriptive statistics in Table 1 show similar trends. Since the linear model is likely only capable of capturing simple correlations between inputs and outputs—alongside exercise-specific harmonics of general movement patterns without considering time contributions—it can serve as a baseline to show whether the additional complexity of the DNN is warranted. The linear model appeared incapable of accurately predicting lateral movements in the Single Leg Stance exercise, possibly due to fewer cyclical movement patterns occurring in this exercise, or because only one tape is used, halving the relevant input signals. The reduced information in the input of the Single Leg Stance trials may also explain why the corresponding DNN model showed a higher MAE compared to the Stepping and Waves exercises. Overall, these baselines confirm that DNNs add value primarily for X/Y, while Z remains input-limited. Figure 5 further shows a pattern of more reliable predictions for smaller CoM displacements, which can likely be explained by the total sample distribution shown in Figure 6: The number of available samples sharply declines for larger displacements, leading to less robust predictions due to insufficient training data for uncommon postural configurations, such as the configuration transitions occurring at the start and end of a trial in Figure 3.

As shown in Table 2, the DNN models exhibited high prediction accuracy for X/Y-axes. For the Z-axis, however, prediction errors were comparable to the movement range, reflecting a lower signal-to-noise ratio in the vertical direction. Despite limitations in tracking vertical CoM displacement, the system’s practical usefulness remains high. For standard forward-facing balance exercises, stability and postural recovery are predominantly governed by anterior-posterior (X) and mediolateral (Y) sway. Accurate tracking of these horizontal components is largely sufficient for detecting movement asymmetries and providing pertinent feedback; thus, the inability to reliably track vertical changes does not critically undermine the system’s core utility for balance training. To better gauge performance, the RMSE was compared with the dataset’s SD, which corresponds to the RMSE of a static prediction of the mean position without considering the input. An RMSE lower than the SD indicates that the model captures underlying patterns beyond simple data variability and outperforms the static predictor. This comparison indicates that the Y-axis models outperform the X-axis models not only in absolute terms, but even more so when viewed in relation to the variance in the observations. The $R^{2}$ values suggest that lateral CoM displacement was most reliably predictable, sagittal displacement less robustly so, and vertical displacement only weakly captured. However, $R^{2}$ should be interpreted with caution in this setting because the models were nonlinear DNNs evaluated on held-out test data. In nonlinear model evaluation, $R^{2}$ alone is insufficient to demonstrate model validity and should be supplemented by additional performance measures [39]. Accordingly, we primarily interpreted model performance using RMSE relative to the reference SD and the mean prediction error, which more directly reflect practical prediction quality.

The main limitation of our dataset is its focus on standard input configurations and movements. The resulting lack of variance in movement errors compromises the models’ predictive accuracy and statistical robustness when applied to non-standard exercise executions. It is also important to note that the orientation of the front and rear tape segments was derived using the Vicon motion capture system. Therefore, this proof-of-concept still relies on a laboratory-assisted sensing configuration rather than exclusively on onboard inertial sensors. This likely constitutes an optimal case regarding the achievable orientation accuracy from orientation sensing devices alone. Deployment in the field would therefore require a prior assessment of potential sensors (such as IMUs with sensor fusion capabilities) to explore the tradeoff between accuracy, cost, and complexity. Another major limitation of our dataset is its restriction to young, healthy participants. Older adults and clinical populations typically exhibit different movement strategies, slower movement velocities, and a greater reliance on external support (e.g., handrails), which affect base kinematics. Consequently, the current models may struggle to generalize to these groups. Applying this system to clinical or geriatric fall-prevention programs will require retraining or at least retesting the models on a more diverse dataset that captures these specific pathological or age-related movement profiles. As movement patterns in clinical or elderly populations are expected to differ significantly, the external validity of our findings for these target domains remains strictly limited until such retraining is complete. Furthermore, since this was intended as a proof of concept, no larger investigation of different model architectures and input variations was performed. For example, it is unclear whether all inputs are needed or how accurate the signals must be to maintain an acceptable prediction error range. Specifically, we did not exhaustively benchmark our heuristic Encoder–LSTM–Decoder against state-of-the-art models. It therefore remains unclear whether the observed performance stems from the architecture itself or the rich input representation. Future work must benchmark this setup against architectures such as Temporal Convolutional Networks (TCNs), GRUs, and Transformers. A further limitation is the trial-level split within participants, which precludes testing of generalization to unseen individuals. Because data from the same individuals are included in both the training and test sets, this setup reduces intrinsic variability and risks subject-specific overfitting, yielding optimistically biased performance. Thus, the model currently serves only as a baseline proof-of-concept; future studies must employ strict leave-one-subject-out cross-validation to rigorously assess true real-world generalizability. Should this investigation indicate diminished accuracy for unseen individuals, a subject-specific model calibration in an initial supervised training session may be required before allowing autonomous training in real-life settings. Future research should therefore focus on eliminating these limitations. Possible improvements include: (1) covering a larger configuration space by training one model for multiple different exercises; (2) comparing different model architectures to identify which is best suited for the task; (3) experimenting with strategies to augment model performance, such as by detecting zero velocity points at which prediction is more reliable (similar to [40]), after which more dynamic phases between these points could be filled using both forward and backward propagation; (4) performing input ablation testing to identify a minimal set of required inputs; and (5) repeating the study with only IMU-based orientation data to gauge robustness to reduced input accuracy and improve the economical viability of the resulting CoM feedback system. Collectively, these steps would clarify the trade-offs between sensor fidelity, model complexity, and deployment practicality.

Nevertheless, the current model provides adequate predictions for lateral CoM position. By contrast, predictions were less reliable for sagittal and least reliable for vertical CoM predictions: the former warrants further investigation, and the latter is unlikely to be easily reconstructible from base of support kinematics alone. However, for practical use, providing positional feedback within permissible movement ranges and flagging larger deviations as movement errors may already constitute beneficial training feedback. In a practical training setting, this system could drive a digital interface positioned in front of the Sensopro. Users would receive terminal or concurrent visual feedback, such as a cursor representing their CoM on a digital target, allowing them to monitor their mediolateral symmetry and sagittal stability. The system could therefore effectively guide users through autonomous coordination training and enable external progress tracking without the need for constant supervision from a therapist or trainer.

## 5. Conclusions

In this work, we have demonstrated that DNN models can leverage accurate tape kinematic data to estimate CoM kinematics during exercises on the Sensopro Luna Fitness. Our results are promising for X/Y-axis CoM position prediction models, while Z-axis performance remains limited. The trained models demonstrated proof-of-concept feasibility under controlled laboratory conditions to predict CoM X/Y positions, enabling CoM-based postural feedback. However, before these models can be deployed for autonomous coordination training in real-world settings, future research must validate inter-subject generalization and demonstrate efficacy using purely onboard, non-optical sensor configurations to satisfy practical limitations regarding cost-effectiveness and usability. Furthermore, training new models on more diverse datasets could lead to more robust prediction accuracy for less common postural configurations. Taken together, these findings support the feasibility of practical X/Y CoM feedback from tape kinematics during balance exercises.

## Figures and Tables

**Table 1:** Descriptive Statistics: Comparison of Linear Model and Deep Neural Network Performance.

Dataset	Linear Model					DNN Model				
	M	SD	Min	Max	Mdn	M	SD	Min	Max	Mdn
Single-Leg Stance CoM X-Axis	26.4	20.5	5.6	124.6	19.5	23.7	6.1	16.39	66.24	23.27
Stepping CoM X-Axis	26.8	21.1	7.9	126.5	18.0	17.1	3.6	12.0	31.2	15.88
Waves CoM X-Axis	26.8	21.7	6.5	128.6	20.4	16.1	1.6	12.8	19.5	16.09
Single-Leg Stance CoM Y-Axis	122.6	8.1	86.3	135.8	124.5	31.3	6.0	21.91	50.57	29.44
Stepping CoM Y-Axis	7.2	2.1	4.5	13.4	6.5	7.8	2.1	5.4	16.9	7.1
Waves CoM Y-Axis	5.2	1.7	2.3	10.9	4.9	4.4	0.9	3.3	8.29	4.1
Single-Leg Stance CoM Z-Axis	25.3	13.2	9.6	78.8	21.4	33.5	2.9	26.2	39.9	33.6
Stepping CoM Z-Axis	21.5	10.3	10.0	63.9	18.1	35.7	3.8	26.4	45.2	35.9
Waves CoM Z-Axis	23.7	8.5	10.8	60.5	20.8	34.7	4.8	28.3	59.4	33.6

*Note.* Values represent mean absolute error (MAE) statistics in millimeters. COM = center of mass; M = mean; SD = standard deviation; Min = minimum; Max = maximum; Mdn = median. Linear Model = linear regression model; DNN = deep neural network.

**Table 2:** Statistical evaluation of prediction errors between reference and predicted center-of-mass (CoM) values. The range of reference values is also listed to provide insight into the central density during exercise execution.

Dataset	Error			Reference		
	M	$R^{2}$	RMSE	SD	Min	Max
Single-Leg Stance CoM X-Axis	7.8	0.58	13.5	20.9	−129.3	389.1
Stepping CoM X-Axis	−1.0	0.30	15.7	18.7	−70.2	392.3
Waves CoM X-Axis	−0.4	0.45	10.5	14.1	−79.5	198.5
Single-Leg Stance CoM Y-Axis	−4.7	0.74	15.9	30.9	−283.4	271.6
Stepping CoM Y-Axis	0.0	0.78	13.8	29.3	−110.4	135.5
Waves CoM Y-Axis	0.4	0.68	4.8	8.5	−64.3	101.7
Single-Leg Stance CoM Z-Axis	−2.3	0.11	12.9	13.7	−75.2	111.3
Stepping CoM Z-Axis	17.6	0.35	42.4	52.6	−157.5	159.4
Waves CoM Z-Axis	5.7	0.38	27.0	34.3	−97.8	275.8

*Note.* Values represent prediction error and reference statistics in millimeters, except for $R^{2}$, which is unitless. COM = center of mass; M = mean; $R^{2}$ = coefficient of determination; RMSE = root mean square error; SD = standard deviation; Min = minimum; Max = maximum.

## Data Availability

The raw data supporting the conclusions of this article will be made available by the authors on reasonable request. Both the code for the neural network models and the corresponding weights will be made available on an online repository [38].

## Appendix A. Exercises

The selection of exercises for this study was primarily informed by the need to capture a broad spectrum of movement characteristics and various challenges to postural control. We specifically identified three exercises—Single Leg Stance, Stepping in Place, and Waves (marked with asterisks in Table A1)—for neural network training because they represent distinct biomechanical profiles, ranging from static balance tasks to dynamic movements with varying degrees of symmetry and frequency.

**Table A1:** The complete list of recorded exercise types: Exercise types performed by participants on the Sensopro Luna. Exercises 1–3 (marked with asterisks) were selected for neural network training based on their distinct movement characteristics.

ID	Exercise	Description
1	Single Leg Stance *	Challenging balance task
2	Stepping * in Place	Slow, asymmetrical movements
3	Waves *	Fast, symmetrical movements
4	Bouncing	Slow, symmetrical movements
5	Sprinting	Fast, asymmetrical movements
6	Lunges	Repeated asymmetrical knee flexions
7	Squats	Repeated symmetrical knee flexions
8	Sideways	Balancing on the two tapes with feet oriented laterally (i.e., both feet pointing to the right)
